# AI-Driven Tacrolimus Dosing in Transplant Care: Cohort Study

**DOI:** 10.2196/67302

**Published:** 2025-09-02

**Authors:** Mingjia Huo, Sean Perez, Linda Awdishu, Janice S Kerr, Pengtao Xie, Adnan Khan, Kristin Mekeel, Shamim Nemati

**Affiliations:** 1 Department of Electrical and Computer Engineering, University of California San Diego, La Jolla, CA, United States; 2 Department of Surgery, University of California San Diego, La Jolla, CA, United States; 3 Skaggs School of Pharmacy and Pharmaceutical Sciences, University of California San Diego, La Jolla, CA, United States; 4 Department of Medicine, University of California San Diego, 9500 Gilman Drive, La Jolla, CA, 92093-0881, United States, 1 4058504751

**Keywords:** organ transplantation, machine learning, clinical decision-making, tacrolimus, personalized medicine

## Abstract

**Background:**

Tacrolimus forms the backbone of immunosuppressive therapy in solid organ transplantation, requiring precise dosing due to its narrow therapeutic range. Maintaining therapeutic tacrolimus levels in the postoperative period is challenging due to diverse patient characteristics, donor organ factors, drug interactions, and evolving perioperative physiology.

**Objective:**

The aim of this study is to design a machine learning model to predict the next-day tacrolimus trough concentrations (C0) and guide dosing to prevent persistent under- or overdosing.

**Methods:**

We used retrospective data from 1597 adult recipients of kidney and liver transplants at UC San Diego Health to develop a long short-term memory (LSTM) model to predict next-day tacrolimus C0 in an inpatient setting. Predictors included transplant type, demographics, comorbidities, vital signs, laboratory parameters, ordered diet, and medications. Permutation feature importance was evaluated for the model. We further implemented a classification task to evaluate the model’s ability to identify underdosing, therapeutic dosing, and overdosing. Finally, we generated next-day dose recommendations that would achieve tacrolimus C0 within the target ranges.

**Results:**

The LSTM model provided a mean absolute error of 1.880 ng/mL when predicting next-day tacrolimus C0. Top predictive features included the recent tacrolimus C0, tacrolimus doses, transplant organ type, diet, and interactive drugs. When predicting underdosing, therapeutic dosing, and overdosing using a 3-class classification task, the model achieved a microaverage *F*_1_-score of 0.653. For dose recommendations, the best clinical outcomes were achieved when the actual total daily dose closely aligned with the model’s recommended dose (within 3 mg).

**Conclusions:**

Ours is one of the largest studies to apply artificial intelligence to tacrolimus dosing, and our LSTM model effectively predicts tacrolimus C0 and could potentially guide accurate dose recommendations. Further prospective studies are needed to evaluate the model’s performance in real-world dose adjustments.

## Introduction

### Background

Tacrolimus is a mainstay of immunosuppressive therapy for patients undergoing solid organ transplants. Despite widespread use and health care provider comfort in managing tacrolimus dosing in the posttransplant period, maintaining the tacrolimus trough concentrations (C0) within the narrow therapeutic range comes with its own set of challenges. In the immediate postoperative period, maintenance of tacrolimus C0 within the therapeutic range is challenging due to baseline patient characteristics, donor organ characteristics, drug-drug interactions, and evolving perioperative physiology resulting in variable day-to-day exposures [1-4]. This variability and, more so, the time out of therapeutic range put patients undergoing solid organ transplants at increased risk of both the development of de novo donor-specific antibodies (dnDSA) from persistent underdosing and tacrolimus toxicity from significantly supratherapeutic levels [5]. Given the risk that inappropriate dosing poses to patients undergoing transplants in terms of organ rejection and tacrolimus toxicity, a considerable amount of time and effort is spent by clinicians to determine and adjust a patient’s dose.

### Challenges and Study Objective

There have been several efforts to reduce this burden on clinicians and improve the time in therapeutic range for recipients of solid organ transplants by using both clinical and genetic variables in the form of publicly available population pharmacokinetics and machine learning models to predict dose-adjusted tacrolimus C0 and recommend optimal tacrolimus dosages [6-11]. However, some of these models require genotyping (ie, CYP3A5 single nucleotide polymorphisms [SNPs]), which may not be widely available in all institutions [6]. In addition, user interface and data requirements for commercially available third-party vendors limit generalizability and practicality for use in the inpatient setting [7]. Methods based on the area under the concentration-time curve require multiple concentration measurements at different time points, making them resource intensive [8,9]. Some studies attempted to predict the optimal stable tacrolimus dosage directly, but this approach is flawed because the “stable” dose may be reached weeks or months after the transplant, during which dnDSA may develop [10,11].

In this work, we describe the development of a machine learning model using electronic health record (EHR) data from Epic Systems to predict the next-day tacrolimus C0 and guide dosing via a recommendation system for clinicians to prevent persistent over- or underdosing in the inpatient setting. Our model accurately predicts tacrolimus C0 by using comprehensive clinical features from a patient’s medical history, including transplant type, demographics, comorbidities, vital signs, laboratory parameter results, and medications, resulting in more personalized tacrolimus C0 predictions and dose recommendations.

## Methods

### Study Design and Dataset

We extracted retrospective data for adult recipients of kidney or liver transplants who received inpatient care at UC San Diego Health from January 2016 to May 2024. These dates were selected to ensure a sufficient sample size and standardization of immunosuppressive therapy. We included adult patients (aged ≥18 years) who received their first liver or kidney transplant. We excluded patients who were transplanted with more than one organ or were receiving a second transplant. Data from 2016 to 2023 were randomly split into 90% for the training set and 10% for the validation set, and the test set contained patients from 2024. The intended use of our model is to predict the next-day tacrolimus C0 based on the features extracted from the patients’ EHRs. To make predictions on the tacrolimus C0 for the next day, we require data of both the tacrolimus C0 and dosage for the current day.

### Ethical Considerations

This study was completed in accordance with the STROBE (Strengthening the Reporting of Observational Studies in Epidemiology) guidelines [12] (Checklist 1) and was approved by the institutional review board at the University of California San Diego (protocol 802489). This retrospective study involved secondary analysis of existing EHR data, for which the institutional review board granted a waiver of informed consent. Only the minimum necessary protected health information was accessed and stored on HIPAA (Health Insurance Portability and Accountability Act)–compliant UC San Diego Health servers; the information will be destroyed upon study closure. No compensation was provided.

### Features

We selected 62 variables as features for each patient, including transplant type, demographics, comorbidities, vital signs, standard inpatient laboratory parameters, ordered diet, and medications, all of which were used to train the model. Transplant type identifies the organ received by the patient (eg, liver or kidney) and is represented as a binary variable. As previously mentioned, only patients who received a single transplant of one type were included. Demographics include age, gender, race, weight, and height. Medications for inpatients were recorded each time they were administered, with timestamps and dose details. This allowed us to calculate the daily dose for each medication. Our medication list included tacrolimus and other drugs known to interact with tacrolimus [13], with a comprehensive list provided in Multimedia Appendix 1. This list includes a total of 12 medications. We recorded the daily tacrolimus dosage in mg, excluding the tacrolimus extended-release dosage, which is rarely used at our institution, particularly for inpatient admissions and in the immediate postoperative period. At our institution, tacrolimus levels are measured using a chemiluminescent microparticle immunoassay on whole blood samples. Any level <2 ng/mL is reported as “<2 ng/mL,” and we used 1 ng/mL to represent data in this range. For other medications, we used a binary variable to indicate whether the patient took the medication within the 3 days prior to the prediction date, accounting for the cumulative drug effect. Vital signs and standard inpatient laboratory parameters were recorded using the built-in measurement unit. Forward filling was applied for imputation, and if no previous data were available for a patient, the global median of nonmissing data was used. Comorbidities considered included hypertension and diabetes. Diabetes was categorized as type 1, type 2, and others, with each category encoded as a binary variable. For categorical features, we used one-hot encoding.

### Long Short-Term Memory Model

We used a long short-term memory (LSTM) [14-16] model in a multistep forecasting approach to predict daily tacrolimus C0 after transplant. The LSTM model architecture is well suited for handling sequential data, as it is designed to capture long-range dependencies by using memory cells to retain information over time.

**Figure 1.**
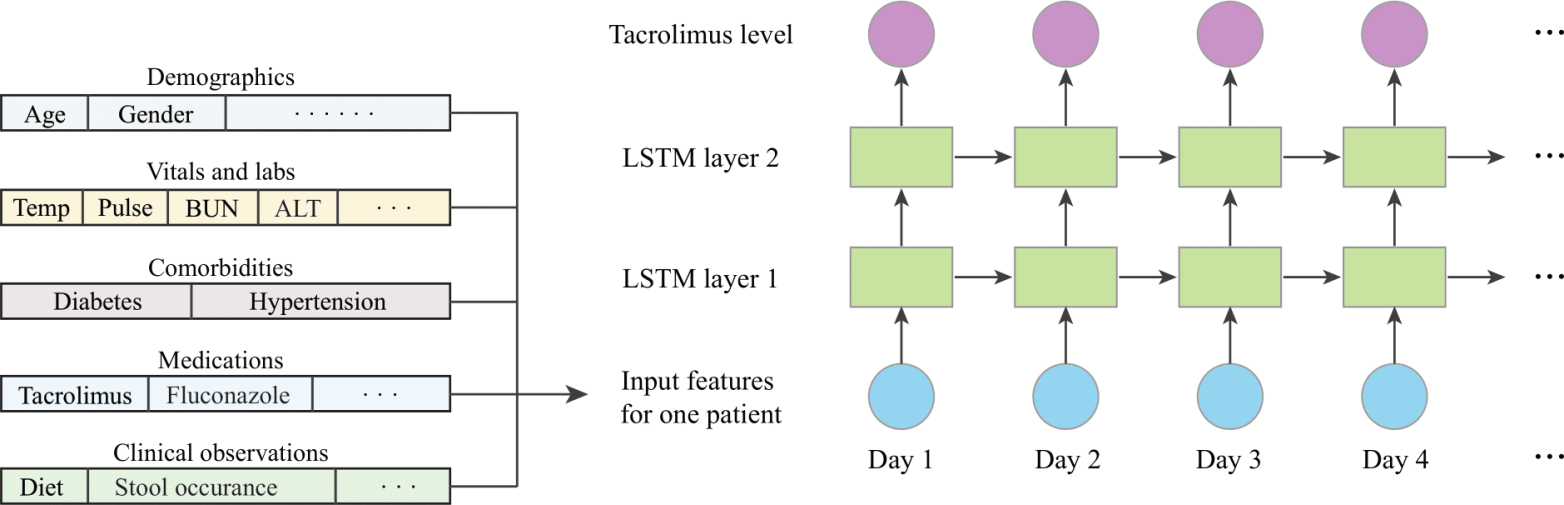
Long short-term memory (LSTM) model architecture with detailed feature representation. ALT: alanine aminotransferase; BUN: blood urea nitrogen; Temp: temperature.

For each patient, we modeled a sequence of daily features, where each feature vector represents the clinical and demographic information available for that specific day. The LSTM model processes this sequence chronologically, leveraging the information from all preceding days to predict the tacrolimus C0 for each subsequent day. Importantly, when predicting the tacrolimus C0 for a particular day, only the features from previous days were considered, ensuring that future information was not used and thus aligning with real-world clinical scenarios. This approach allows the model to dynamically incorporate the evolving clinical profile of the patient as more data become available over time, effectively capturing the temporal dependencies inherent in the patient’s posttransplant clinical course progression. The architecture of our LSTM model is illustrated in Figure 1.

Studying the influence of different features is necessary for clinicians to understand the potential relationship with predicted tacrolimus concentrations. To evaluate the feature importance of the model, we used permutation importance (also known as model class reliance) [17]. This was obtained by randomly shuffling the values of each feature and observing the impact on the model’s predictive performance, thereby quantifying the reliance of the model on each specific feature. A higher performance decrease indicates the feature is more important.

### Data Processing, Training, and Hyperparameters

Feature normalization was performed to scale values into the (0,1) range. The LSTM models were trained using the Adam optimizer. Regularization was applied to minimize overfitting and enhance generalizability. A hyperparameter search was conducted for the LSTM model by optimizing the size and number of LSTM layers, dropout rate, learning rate, and the regularization parameter using 5-fold cross-validation on the training cohorts using Bayesian optimization [18]. The loss function used in the hyperparameter search is mean squared error (MSE). In addition, other model architectures (Multimedia Appendix 2) were trained and compared, and the LSTM model was selected as the best-performing model based on the MSE on the validation set. All experiments were performed using Jupyter Notebook 6.5.6 (Jupyter) in a secure cloud environment within AWS SageMaker (Amazon Web Services). The model was implemented using Python (version 3.8.18), with data preprocessing performed using NumPy (version 1.24.4) and pandas (version 2.0.3), and model training was conducted using PyTorch (version 2.0.0).

### Metrics for Tacrolimus C0 Prediction

Mean absolute error (MAE) and mean absolute percentage error (MAPE) were calculated on the test set to evaluate the performance of the models. MAE is calculated as the average of the absolute differences between the predicted and actual concentrations. MAPE is calculated as the average of the absolute differences between the predicted and actual concentrations divided by the actual concentrations and is expressed as a percentage.

### Tacrolimus Underdosing, Overdosing, or Therapeutic Dosing Concentrations Classification

We designed a 3-class classification task to assess the model’s ability to predict underdosing, overdosing, or therapeutic dosing, based on our institution’s defined therapeutic window for patients undergoing kidney transplant with good kidney function and patients undergoing liver transplant with high risk factors for rejections across different posttransplant periods. The target tacrolimus C0 for patients undergoing kidney transplant are 10 to 13 ng/mL during the first 0 to 3 months after the transplant and 8 to 10 ng/mL after 3 months. For patients undergoing liver transplant, the target concentrations are 7 to 10 ng/mL within the first year after the transplant and 5 to 7 ng/mL 1 year after the transplant. The models were evaluated using precision, recall, and *F*_1_-scores. Given the multiclass nature of the task, we report both the microaverage (equal weight to each instance) and macroaverage (equal weight to each class) for precision, recall, and *F*_1_-scores to represent the overall and per-class performance, respectively.

Due to the limited data available on underdosing and overdosing cases, we implemented an oversampling strategy [19] to balance the training dataset. Specifically, we duplicated instances with tacrolimus C0 below 5 ng/mL or above 13 ng/mL 3 times, resulting in a total of 4 instances for each of these cases during training. This approach aims to enhance the model’s ability to recognize these critical yet underrepresented cases. We report the classification results for both the original dataset (uniform sampling) and the oversampled dataset, providing a comprehensive evaluation of the model’s performance under different training conditions.

### Dose Recommendation

We calculated the recommended dose based on the predicted tacrolimus C0. This was achieved by considering all possible dose options within a 15-mg range for a single dose, with increments of 0.5 mg, administered twice daily. We ensured that the difference between the morning and evening doses was limited to 0 mg, 0.5 mg, or 1 mg, with the additional restriction that the recommended daily dose remains within 1.5 times the previous day’s dose. This threshold was chosen after discussion with our multidisciplinary group, which included transplant pharmacists and health care providers, to increase the usability of this recommendation system. The recommended dose selected was the one that resulted in the best predicted C0, specifically the mean value within the therapeutic window defined for the patient.

We evaluated the effectiveness of the recommended dose by defining a reward function, R(.), using the formula from Lin et al [20]. It translates the measured outcomes into a continuous reward using the following function:

$R(x)=\frac{c}{1+e^{-(x-b_{lower})}}-\frac{c}{1+e^{-(x-b_{upper})}}$

Here, x is the tacrolimus C0, and $b_{lower}$ and $b_{upper}$ are the lower and upper bounds, respectively, given by our institution’s defined therapeutic windows. The constant c scales the reward function appropriately within the range [0,1]. See Multimedia Appendix 3 for the shape of the reward function. This function yields a high reward close to 1 when x is within the target range, and the reward gradually decreases to 0 as x moves outside this range.

## Results

### Patient Characteristics

**Figure 2.**
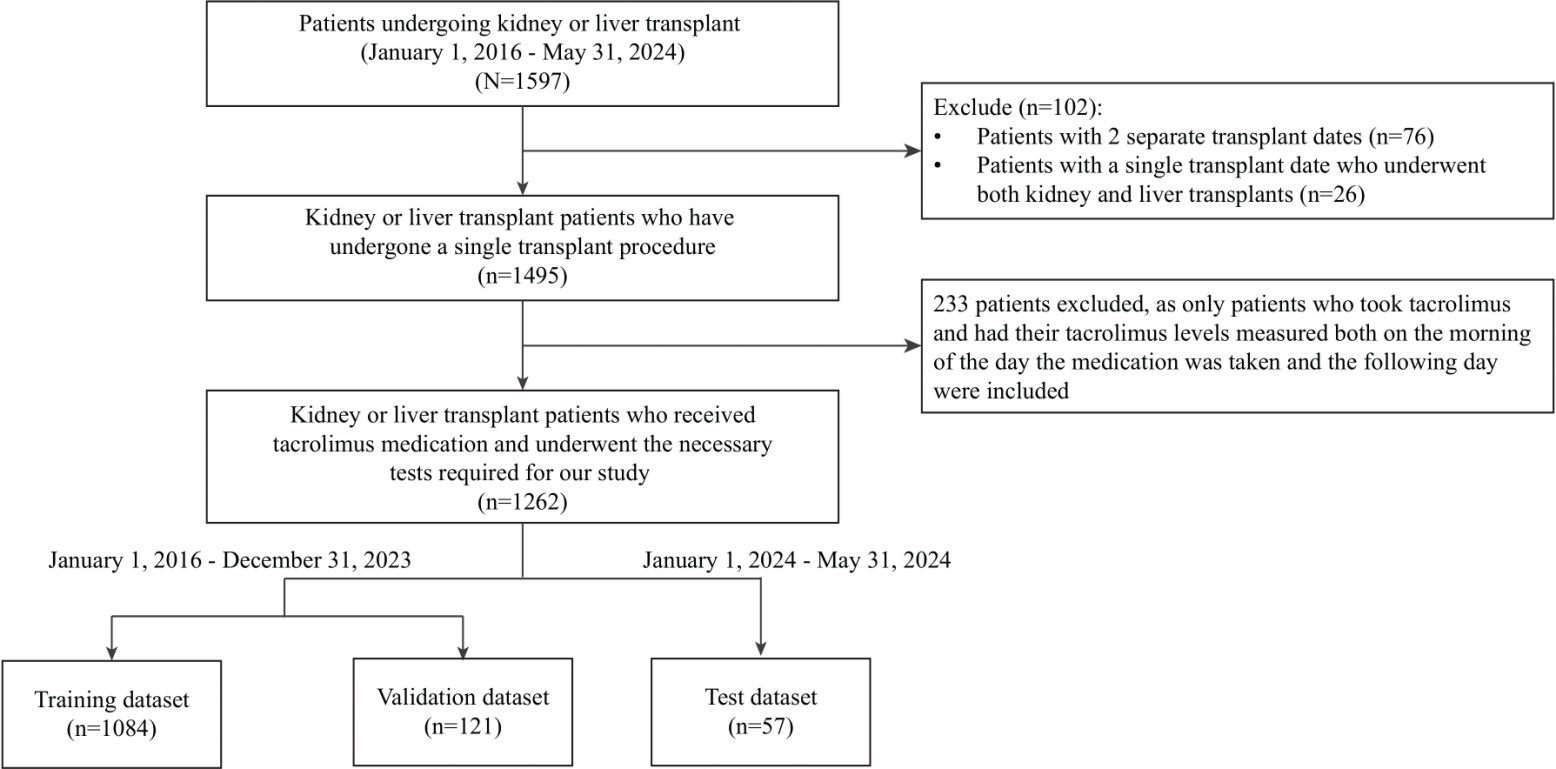
Patient selection and data processing flowchart.

We identified 1597 patients undergoing transplants who received at least one kidney or liver transplant between January 1, 2016, and May 31, 2024. Of these, 1495 patients received only one type of transplant, with 1033 patients undergoing kidney transplant and 462 patients undergoing liver transplant. The cohort was further refined to 1262 patients after ensuring that they had consistent patient records, including taking tacrolimus, having their tacrolimus C0 measured on 2 consecutive mornings, and administering the medication after the first measurement. This includes 825 kidney recipients and 437 liver recipients. A detailed flowchart of the patient selection process is provided in Figure 2.

The final dataset consisted of 9601 tacrolimus C0 measurements to be predicted, with 4602 from kidney recipients and 4999 from liver recipients. On average, patients undergoing kidney transplant contributed 5.6 predictions and patients undergoing liver transplant contributed 11.4 predictions to the entire cohort. The median daily tacrolimus dose was 5.0 mg for both kidney and liver recipients. The median (IQR) tacrolimus concentration was 8.4 (6.4‐10.8) ng/mL for kidney and 7.5 (5.5‐9.7) ng/mL for liver recipients. The median (IQR) age was 58 (47-65) years, with 47% of kidney recipients and 44.8% of liver recipients identifying as White. Notably, hypertension was more prevalent among kidney recipients (86.5%) than liver recipients (54%), while the prevalence of diabetes was similar between the two groups (51.9% for kidney and 49.2% for liver recipients). The dataset spanned a median (IQR) of 15 (3-123) days after the transplant for kidney recipients and 19 (5-113) days for liver recipients. See Table 1 for a detailed description of patient characteristics.

**Table 1.** Patient characteristics.

	Kidney recipients (n=825; 4602 days)	Liver recipients (n=437; 4999 days)
Age (years), median (IQR)	58 (47-65)	58 (47-65)
Gender (man), n (%)	540 (65.5)	258 (59.0)
Weight (kg), median (IQR)	77.2 (67.0‐90.1)	73.3 (61.1‐87.5)
Height (cm), median (IQR)	170.0 (160.7‐177.0)	167.6 (160.0‐176.5)
Time after transplant (day), median (IQR)	15 (3-123)	19 (5-113)
Race, n (%)		
American Indian or Alaska Native	5 (0.6)	0 (0)
Asian	134 (16.2)	20 (4.6)
Black or African American	79 (9.6)	12 (2.7)
Native Hawaiian or other Pacific Islander	7 (0.8)	2 (0.5)
Other race or mixed race	206 (25.0)	201 (46.0)
White	388 (47.0)	196 (44.9)
Skip or prefer not to answer or not indicated	6 (0.7)	6 (1.4)
Ethnicity, n (%)		
Hispanic or Latino	364 (44.1)	214 (49.0)
Non-Hispanic or Latino	460 (55.8)	218 (49.9)
Skip or prefer not to answer or not indicated	1 (0.1)	5 (1.1)
Comorbidities, n (%)		
Hypertension	714 (86.5)	236 (54.0)
Diabetes	428 (51.9)	215 (49.2)
Medications		
Daily tacrolimus (mg), median (IQR)	5.0 (2.0‐7.0)	5.0 (2.5‐8.0)
Use of mycophenolate mofetil, n (%)	366 (44.4)	238 (54.5)
Daily mycophenolate mofetil (mg), median (IQR)	1500 (1000‐2000)	1500 (1000‐2000)
Use of antithymocyte globulin, n (%)	154 (18.7)	4 (0.9)
Daily antithymocyte globulin (mg), median (IQR)	75 (100-125)	75 (50-100)
Use of sirolimus, n (%)	8 (1.0)	7 (1.6)
Daily sirolimus (mg), median (IQR)	1.0 (0.5‐2.0)	1.0 (1.0‐1.5)
Labs		
Tacrolimus (ng/mL), median (IQR)	8.4 (6.4‐10.8)	7.5 (5.5‐9.7)

### Performance

The LSTM model demonstrated an MAE of 1.880 ng/mL in predicting the next day’s tacrolimus C0, taking both patients undergoing kidney and liver transplants into consideration. If analyzed separately, the MAE was 1.973 ng/mL for patients undergoing kidney transplant and 1.744 ng/mL for patients undergoing liver transplant. In addition, we computed the MAE if the measured levels were below, within, and above the tacrolimus target window, which was 1.427, 1.521, and 4.365 ng/mL, respectively. When evaluated using the MAPE, the model achieved an average rate of 24.3% for both patients undergoing kidney and liver transplants, with a MAPE of 23.8% for patients undergoing kidney transplant and 25.0% for patients undergoing liver transplant. We observed a 21% decrease in the MAE when evaluating predictions from days 5 to 10 after the transplant compared to days 1 to 5. The final hyperparameters were 2 LSTM layers with a hidden state size of 48, a dropout rate of 0.1, a learning rate of 2e^–3^, a batch size of 16, and an L2 regularization (weight decay) of 1e^–5^.

The model’s capability to predict underdosing or overdosing was evaluated using the target concentration ranges in a 3-class classification task. As shown in Table 2, the model achieved an overall microaverage *F*_1_-score of 0.653. The *F*_1_-score for predicting underdosing was notably higher at 0.795, reflecting the model’s strong ability to forecast underdosing.

**Table 2.** Three-class classification results with an oversampling of underdosing and overdosing cases during training.

	Underdosing	Therapeutic	Overdosing	Macroaveraged	Microaveraged
Precision	0.851	0.427	0.405	0.561	0.673
Recall	0.746	0.571	0.357	0.558	0.642
*F*_1_-score	0.795	0.489	0.380	0.555	0.653

### Important Features for Tacrolimus C0 Prediction

Here, we report on the top 10 most important features for the proposed LSTM model. The feature importance analysis revealed that the most recent tacrolimus C0 (tacro_level_today) is the most critical factor in predicting future concentrations, indicating that the model heavily relies on the latest available concentration. The type of organ transplanted (eg, kidney) and variables related to the tacrolimus dose—specifically, tacro_dose_prev_16_24, tacro_dose_0_8, and tacro_dose_16_24, which represent the dose administered 16 to 24 hours earlier, 0 to 8 hours on the current day, and 16 to 24 hours on the current day, respectively—also demonstrate significant importance. Medications such as ketoconazole, fluconazole, posaconazole, and prednisone, which are known for their interactions with tacrolimus, are also key predictors, aligning with clinical expectations. The number of recorded meals (ie, standard diet) also contributes meaningfully to the model’s predictions, potentially due to the impact of nutritional status on drug absorption and metabolism [21]. Overall, the analysis highlights the model’s reliance on recent tacrolimus C0, tacrolimus doses, organ type, diet, and drug interactions in predicting tacrolimus C0.

### Dose Recommendation

**Figure 3.**
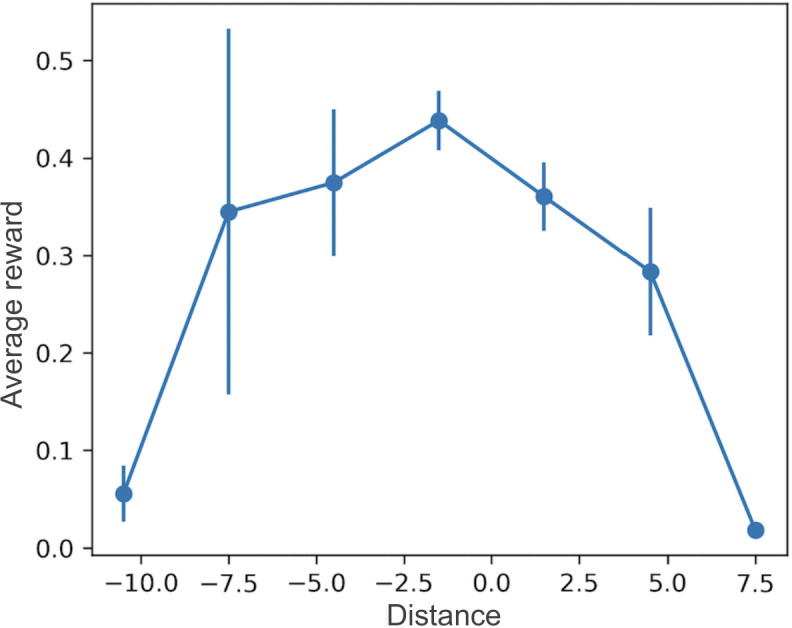
Association between the difference in actual and recommended daily doses (distance) and average reward (mean and SE).

We analyzed the relationship between the difference in actual and recommended daily doses and the corresponding reward in terms of means and SEs (Figure 3). We observed that when this difference approaches zero, the average rewards are the highest, suggesting that when clinicians select doses closer to the recommended values, the tacrolimus C0 are optimal. Conversely, as the difference increases, the reward tends to decline. These findings suggest that our recommended algorithm, based on the level prediction model using LSTM, provides reasonable and useful guidance for tacrolimus dosing.

## Discussion

### Principal Findings

We presented a data-driven clinical decision support tool for optimizing posttransplant immunosuppressive therapy with the potential to improve patient outcomes. In comparison to other previously described attempts that use Bayesian modeling or machine learning methods, we have accomplished the development of an accurate prediction model using only clinical variables available within the EHR. The LSTM model is designed to process sequential data, capturing long-term dependencies that are crucial in clinical settings where patient responses to medication evolve over time [22].

In using institutional data for model development, we recognized that SEs associated with daily laboratory measurements likely impact prediction accuracy. This error is present in all tacrolimus C0 measurements regardless of institution; however, previous studies aiming to predict daily tacrolimus C0 measurements for patients undergoing solid organ transplants fail to address this [6,23]. At our institution, precision and accuracy rates of daily quality control C0 measurements are both 5%. This SE, in combination with the variability of tacrolimus administration times and tacrolimus C0 lab draw times, likely plays a significant role in the accuracy of our prediction model and may prove to be a challenge when moving to real-world implementation of prediction and dose recommendation models.

In practice, dosing adjustments are based on a clear and comprehensive clinical picture, considering possible medication interactions and patient physiology. For the experienced and thorough health care provider, these adjustments are made fairly easily, whereas an inexperienced or nonspecialist health care provider adjusting these medications in an inpatient setting may experience difficulty consistently maintaining tacrolimus C0 measurements within the therapeutic window. Our dosing recommendation system offers a tool for health care providers to guide dosing adjustment, and next steps for this study include clinical validation. Previous dosing recommendations have largely been focused on initial dosing strategies, based on genotypes and clinical variables [24,25], but do not give daily recommendations. For large-volume transplant centers, these recommendations can save considerable time and effort, particularly with complex patients.

### Comparison With Prior Work

Previous studies have developed predictive models based on genotype data, highlighting the potential for incorporating genetic information into future models to further enhance prediction accuracy [6]. We opted not to incorporate this for several reasons: the prediction and dose recommendation models were developed with the intention of clinical implementation, and health care providers at our institution do not routinely use genomics in their practice. Min et al [6] reported on 3 tacrolimus C0 prediction models using clinical variables alone, genomic variables alone, and a combination of clinical variables with CYP3A5 SNPs. The hybrid model performed the highest with a log-transformed MSE of 0.61 for their LASSO (least absolute shrinkage and selection operator) model. Meanwhile, our model using clinical variables alone achieves a log-transformed MSE of 0.10, outperforming the LASSO model. This suggests that while CYP3A5 SNPs may be important in determining initial dosing regimens, they may be less important when considering day-to-day tacrolimus C0 and dosing, although this may also be due to differences in the models used for our predictions.

### Limitations

Hospital admissions following the index transplant typically occur due to significant illnesses, particularly infections, at which point pharmacokinetics are difficult to predict and lead to higher MAEs. Conditions such as diarrhea, infections (particularly those requiring azole antifungals), emesis, presence of continuous tube feeding, periods of fasting states, and additional surgeries that lead to bleeding further complicate accurate dosage prediction. We conducted a manual review of patient charts where the model showed significant inaccuracies and found that many relevant factors are better documented in clinical notes than in flow sheets and flowcharts. This indicates that using natural language processing techniques to extract this information could improve prediction accuracy, highlighting a promising area for future research [26,27]. Due to the frequency with which patients who have undergone solid organ transplants are admitted to the hospital, this population may benefit from the development of their own unique model, that is, separate models for the immediate postoperative period and subsequent readmissions. In addition, because each patient contributes to multiple predictions and later-stage predictions tend to be more accurate, the overall MAE may be biased depending on the distribution of predictions across different stages of hospitalization.

Given the variability in patient conditions, some patients may experience a departure from the baseline “steady state” physiology. However, as our model requires input of the current day’s dose and tacrolimus C0, critically ill patients whose tacrolimus C0 is on hold will not be incorporated in the model. Nevertheless, we captured patients who are not critically ill, such as those with delayed graft function [28] or biliary stricture [29]. Therefore, our model can account for moderate deviations from baseline with reasonable accuracy, providing clinicians with a valuable tool for managing various cases. For future research, we consider developing a separate model for nonstable patients and using an ensemble model to jointly predict outcomes for both steady and nonsteady patients.

### Conclusions

By accurately forecasting tacrolimus C0, the proposed LSTM model can provide valuable guidance for dosage adjustments among patients undergoing kidney and liver transplants, with the potential to improve clinical outcomes by maintaining therapeutic drug levels and reducing the risk of underdosing or overdosing. The model performs particularly well in identifying patients likely to be subtherapeutic, suggesting that clinical implementation of the tool may aid in improving metrics, such as time to therapeutic concentration and days spent within the therapeutic window, and in limiting the risk of acute rejection. Integrating genetic data and using natural language processing techniques to capture additional clinical nuances may further refine the model’s accuracy and applicability. Future directions include clinical implementation and assessment of real-time utility of this clinical decision support tool for dosing recommendations.

## Supplementary material

10.2196/67302 Multimedia Appendix 1 List of interactive drugs and their nonempty occurrence rates in the dataset.

10.2196/67302 Multimedia Appendix 2 A comparison of the long short-term memory (LSTM) model with baseline models.

10.2196/67302 Multimedia Appendix 3 The reward function for target range (10,13) as an example.

10.2196/67302 Checklist 1 STROBE guidelines checklist.

10.2196/67302 Checklist 2 CREMLS checklist.

## Abbreviations

C0: trough concentrations
dnDSA: de novo donor-specific antibodies
EHR: electronic health record
HIPAA: Health Insurance Portability and Accountability Act
LASSO: least absolute shrinkage and selection operator
LSTM: long short-term memory
MAE: mean absolute error
MAPE: mean absolute percentage error
MSE: mean squared error
SNP: single nucleotide polymorphism
STROBE: Strengthening the Reporting of Observational Studies in Epidemiology
