# Supplementary material for: AI-Driven Tacrolimus Dosing in Transplant Care: Cohort Study
Source: JMIR AI. 2025 Sep 2;4:e67302. doi: 10.2196/67302 (PMC12404564; doi:10.2196/67302)
Supplement: Multimedia Appendix 1 [file ai-v4-e67302-s001.docx]

Multimedia Appendix 1. List of interactive drugs and their non-empty occurrence rates in the dataset.

|  | **Kidney recipients** | **Liver recipients** |
| --- | --- | --- |
| **Medication Medications that Increase Tacrolimus Levels** | | |
| Calcium Channel Blockers | | |
| *diltiazem, (%)* | 1.1 | 0.7 |
| *verapamil, (%)* | 0.2 | 0.2 |
| *nicardipine, (%)* | 4.5 | 3.0 |
| Antifungals | | |
| *fluconazole, (%)* | 14.7 | 21.9 |
| *ketoconazole, (%)* | 0.0 | 0.9 |
| *voriconazole, (%)* | 2.2 | 2.1 |
| *posaconazole, (%)* | 10.0 | 6.3 |
| Macrolide Antibiotics | | |
| *erythromycin, (%)* | 0.5 | 0.8 |
| **Medications that Decrease Tacrolimus Levels** | |  |
| Anticonvulsants | | |
| *phenytoin, (%)* | 0.1 | 0.0 |
| *carbamazepine, (%)* | 0.1 | 0.1 |
| Antibiotics | | |
| *rifabutin, (%)* | 0.9 | 0.1 |
| Glucocorticoids | | |
| *prednisone, (%)* | 44.2 | 49.5 |
