# Supplementary material for: AI-Driven Tacrolimus Dosing in Transplant Care: Cohort Study
Source: JMIR AI. 2025 Sep 2;4:e67302. doi: 10.2196/67302 (PMC12404564; doi:10.2196/67302)
Supplement: Multimedia Appendix 2 [file ai-v4-e67302-s002.docx]

Figure S1. Summary of prediction performance of different models with uniform sampling during training. This figure compares the performance of five models—XGBoost, Multi-layer Perceptron (MLP), k-nearest neighbors (KNN), Recurrent Neural Network (RNN), and Long Short-Term Memory (LSTM)—using three metrics: Mean Absolute Error (MAE), Mean Absolute Percentage Error (MAPE), and Mean Squared Error (MSE). XGBoost, MLP, KNN use fixed input features consisting of the previous two days' tacrolimus dosage information and the previous day's data for other features. The weights used to compute distance for KNN are derived from a linear regression model. RNN and LSTM, which handle sequential data, utilize the full sequence of data for predictions.


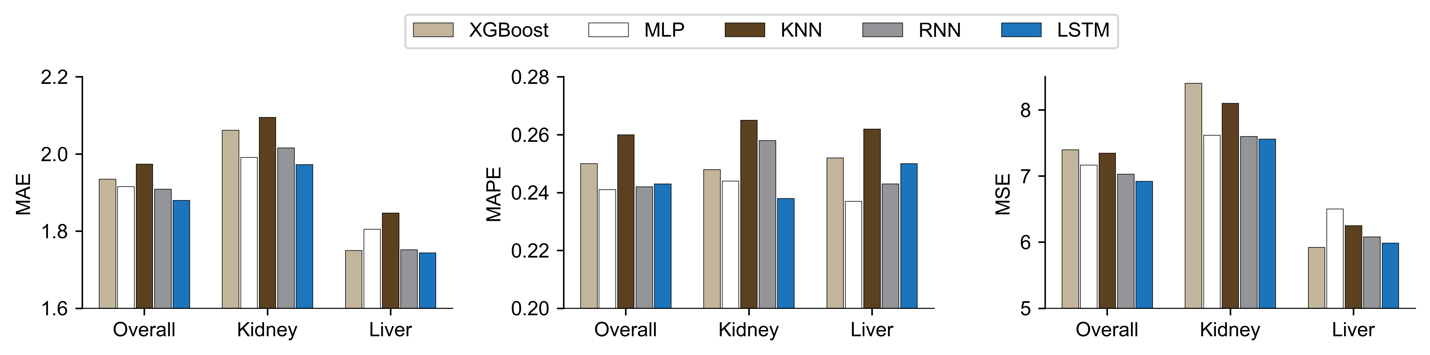


Table S1. 3-class classification results for different models with oversampling of underdosing and overdosing cases during training.

|  |  | **Underdosing** | **Therapeutic** | **Overdosing** | **Macro-averaged** | **Micro-averaged** |
| --- | --- | --- | --- | --- | --- | --- |
| **XGBoost** | Precision | 0.802 | 0.444 | 0.457 | 0.568 | 0.659 |
|  | Recall | 0.807 | 0.480 | 0.381 | 0.556 | 0.660 |
|  | F1 | 0.805 | 0.462 | 0.416 | 0.561 | 0.659 |
| **MLP** | Precision | 0.795 | 0.411 | 0.341 | 0.516 | 0.629 |
|  | Recall | 0.795 | 0.400 | 0.357 | 0.517 | 0.628 |
|  | F1 | 0.795 | 0.405 | 0.349 | 0.517 | 0.629 |
| **KNN** | Precision | 0.811 | 0.430 | 0.422 | 0.555 | 0.655 |
|  | Recall | 0.778 | 0.453 | 0.452 | 0.561 | 0.646 |
|  | F1 | 0.794 | 0.442 | 0.437 | 0.557 | 0.650 |
| **RNN** | Precision | 0.865 | 0.410 | 0.350 | 0.542 | 0.668 |
|  | Recall | 0.757 | 0.532 | 0.333 | 0.541 | 0.635 |
|  | F1 | 0.808 | 0.463 | 0.341 | 0.537 | 0.648 |
| **LSTM** | Precision | 0.851 | 0.427 | 0.405 | 0.561 | 0.673 |
|  | Recall | 0.746 | 0.571 | 0.357 | 0.558 | 0.642 |
|  | F1 | 0.795 | 0.489 | 0.380 | 0.555 | 0.653 |
