# Supplementary figures and images for: AI-Driven Tacrolimus Dosing in Transplant Care: Cohort Study
Source: JMIR AI. 2025 Sep 2;4:e67302. doi: 10.2196/67302 (PMC12404564; doi:10.2196/67302)

Multimedia Appendix 3. The reward function for target range (10,13) as an example.


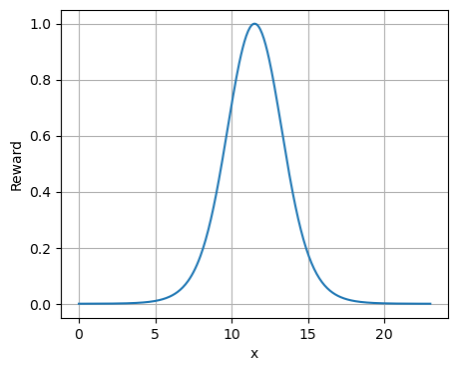

Supplement: Multimedia Appendix 3 [file ai-v4-e67302-s003.docx]
