# Supplementary material for: AI-Driven Tacrolimus Dosing in Transplant Care: Cohort Study
Source: JMIR AI. 2025 Sep 2;4:e67302. doi: 10.2196/67302 (PMC12404564; doi:10.2196/67302)
Supplement: Checklist 2 [file ai-v4-e67302-s005.pdf]

Checklist 2. Illustration of how various articles published in JMIR Publications journals implement each of the CREMLS (Consolidated Reporting of Machine Learning Studies) checklist items.

Item number	Item	Description
**Study details**		
1.1	The medical or clinical task of interest	Maintain tacrolimus trough concentration for kidney/liver transplant patients under in-patient setting.
1.2	The research question	How to use machine learning models to guide tacrolimus dosing to maintain trough concentration.
1.3	Current medical or clinical practice	Clinicians manually decide tacrolimus dosing for inpatients on a daily basis, based on the history of dosing and lab results.
1.4	The known predictors and confounders of what is being predicted or diagnosed	Predictors: Tacrolimus dose and C0 history, transplant organ type, demographics, comorbidities (hypertension, diabetes), vital signs, standard inpatient laboratory parameters, diet, and medications (including interacting drugs).<br><br>Confounders: Standard errors in laboratory measurements.
1.5	The overall study design	Retrospective cohort study using electronic health records (EHR) from kidney and liver transplant patients at UC San Diego Health from 2016 to 2024. Patients from 2016 to 2023 were split into a training set (90%) and validation set (10%), while patients from 2024 were used as the test set.
1.6	The medical institutional settings	UC San Diego Health, covering inpatient kidney and liver transplant recipients.
1.7	The target patient population	Adult kidney and liver transplant recipients (⩾18 years old), excluding multi-organ transplant recipients or patients receiving a second transplant.
1.8	The intended use of the ML model	Predict next-day tacrolimus trough concentration (C0) and provide dose recommendations to maintain therapeutic levels.
1.9	Existing model performance benchmarks for this task	Previous models used Bayesian methods, pharmacokinetic modeling, or genotype-based approaches. Compared to Min et al.'s model (log-transformed MSE of 0.61), the proposed LSTM model achieved a log-transformed MSE of 0.10, outperforming the previous method.
1.10	Ethical and other regulatory approvals obtained	Approved by the Institutional Review Board (IRB) at UCSD (protocol #802489).
**The data**		
2.1	Inclusion or exclusion criteria for the patient cohort	Inclusion: Adult (⩾18 years) kidney and liver transplant recipients.<br><br>Exclusion: Multi-organ transplant recipients or patients receiving a second transplant.

Item number	Item	Description
2.2	Methods of data collection	Retrospective extraction of structured EHR data, including transplant type, demographics, comorbidities, vital signs, standard inpatient laboratory parameters, ordered diet, and medications.<br><br>We require the patient to have their tacrolimus concentrations measured on two consecutive mornings, and get the medication after the first measurement.
2.3	Bias introduced due to the method of data collection used	Bias may arise since we use all previous tacrolimus doses and labs to make predictions, and patients with more observations in the test are better predicted.
2.4	Data characteristics	See Table 1 for detailed data characteristics.
2.5	Methods of data transformation and preprocessing applied	We used one-hot encoding for categorical variables, and normalization of continuous variables to [0,1] range. Also see 3.1 and 3.2 in this table.
2.6	Known quality issues with the data	Missing values in vitals and laboratory parameters.
2.7	Sample size calculation	After data preprocessing, we get 825 kidney recipients with 4602 measurements and 437 liver recipients with 4999 measurements. The entire dataset was used for training, validation, and testing without further sampling.
2.8	Data availability	Not publicly available due to patient privacy regulations.
**Methodology**		
3.1	Strategies for handling missing data	For vitals and laboratory parameters, forward filling was applied for imputation, and if no previous data was available for a patient, the global median of non-missing data was used.
3.2	Strategies for addressing class imbalance	Oversampling of tacrolimus concentrations below 5 ng/mL or above 13 ng/mL three times during training to improve model learning in underrepresented cases.
3.3	Strategies for reducing dimensionality of data	To consider interactive drugs, we used a binary variable to indicate whether the patient took a medication within the three days prior to the prediction date. If we had chosen to use a separate variable for each day, this would have required three variables per medication. This choice effectively reduced dimensionality while considering the influence of interactive drugs.
3.4	Strategies for handling outliers	An outlier detection mechanism was implemented for vital signs and laboratory values during data preprocessing, replacing extreme values with respective upper and lower bounds.
3.5	Strategies for data augmentation	Same as 3.2.
3.6	Strategies for model pretraining	Model was trained from scratch.
3.7	The rationale for selecting the ML algorithm	LSTM was chosen due to its ability to capture temporal dependencies in sequential clinical data. We also trained RNN, MLP, XGBoost, KNN for comparison.

Item number	Item	Description
3.8	The method of evaluating model performance during training	5-fold cross-validation with mean squared error (MSE) as the loss function to tune each model.
3.9	The method used for hyperparameter tuning	Bayesian optimization was used to tune LSTM number of layers, hidden state size, dropout rate, learning rate, batch size and regularization parameters.
3.10	Model's output adjustments	The model's output is tacrolimus C0, and we recommend the doses by enumerating possible doses and choose the doses that can reach target C0.
**Evaluation**		
4.1	Performance metrics used to evaluate the model	• Mean squared error (MSE), Mean absolute error (MAE), mean absolute percentage error (MAPE) for C0 prediction.<br>• Precision, Recall, and F1 scores for classification on under/therapeutic/over-dosing.<br>• Average rewards based on the difference between actual and recommended doses.
4.2	The cost or consequence of errors	Underdosing may lead to organ rejection, while overdosing increases the risk of toxicity.
4.3	The results of internal validation	MAE is 1.783 ng/mL on the validation set, which consists of a randomly sampled 10% of patients from 2016–2023. However, since the model is intended for use in new patients, our paper primarily reports performance on the test set (2024 patients), which is considered as an external data set. See 4.5 for details.
4.4	The final model hyperparameters	LSTM Model:<br><br>layer = 2<br>Hidden state size = 48<br>Dropout rate = 0.1<br>Learning rate = 2e-3<br>Batch size = 16<br>Weight decay (L2 regularization) = 1e-5
4.5	Model evaluation on an external data set	We constructed a test set using 2024 patients as the external data set.<br><br>Results: MAE of 1.880 ng/mL across kidney and liver recipients. F1-score of 0.653 for underdosing/therapeutic/overdosing classification.
4.6	Characteristics relevant for detecting data shift and drift	Patient demographics, medication patterns, post-transplant days, number of past tacrolimus observations, and changes in laboratory protocols could introduce data shift.
**Explainability and transparency**		
5.1	The most important features and how they relate to the outcomes	Top features: Most recent tacrolimus concentration, transplant organ type, previous tacrolimus doses, diet, and interactive drugs (e.g., ketoconazole, fluconazole). See the "Important Features for Tacrolimus Concentration Prediction" subsection for details.

Item number	Item	Description
5.2	Plausibility of model outputs	We analyzed dose recommendations based on designed rewards (See “Dose recommendation” subsection). When clinicians select doses closer to the recommended values, the outcomes improve, as reflected in higher reward. As the deviation increases, the reward declines. These results suggest that our model provides reliable guidance for tacrolimus dosing.
5.3	Interpretation of a model's results by an end user	Global feature importance can be interpreted via permutation importance analysis.<br><br>For a specific prediction, one can use SHAP values to determine the positive or negative impact for each feature. However, SHAP treats features independently, ignoring the dependencies between doses and laboratory values. Therefore, we choose not to disclose SHAP values for specific samples to avoid misleading interpretations.
